# Macrophages promote angiogenesis in human breast tumour spheroids *in vivo*

**DOI:** 10.1038/sj.bjc.6602901

**Published:** 2005-12-13

**Authors:** L Bingle, C E Lewis, K P Corke, M W R Reed, N J Brown

**Affiliations:** 1 Microcirculation Research Group, University of Sheffield Medical School, Sheffield, S10 2RX, UK; 2 Tumour Targeting Group, School of Medicine & Biomedical Sciences, University of Sheffield Medical School, Sheffield, S10 2RX, UK

**Keywords:** angiogenesis, macrophage, dorsal skinfold chamber model, vascular endothelial growth factor (VEGF)

## Abstract

An *in vivo* model has been established to study the role of macrophages in the initiation of angiogenesis by human breast tumour spheroids *in vivo*. The extent of the angiogenic response induced by T47D spheroids implanted into the dorsal skinfold chamber in nude mice was measured *in vivo* and compared to that induced by spheroids infiltrated with human macrophages prior to implantation. Our results indicate that the presence of macrophages in spheroids resulted in at least a three-fold upregulation in the release of vascular endothelial growth factor (VEGF) *in vitro* when compared with spheroids composed only of tumour cells. The angiogenic response measured around the spheroids, 3 days after *in vivo* implantation, was significantly greater in the spheroids infiltrated with macrophages. The number of vessels increased (macrophages *vs* no macrophages 34±1.9 *vs* 26±2.5, *P*<0.01), were shorter in length (macrophages *vs* no macrophages 116±4.92 *vs* 136±6.52, *P*<0.008) with an increased number of junctions (macrophages *vs* no macrophages 14±0.93 *vs* 11±1.25, *P*<0.025) all parameters indicative of new vessel formation. This is the first study to demonstrate a role for macrophages in the initiation of tumour angiogenesis *in vivo*.

Angiogenesis, the development of new blood vessels from an existing vascular network, is a prerequisite for tumour growth beyond 2 mm in diameter, as it ensures the adequate supply of oxygen and nutrients for tumour cell expansion and spread (Folkman, 1971; Skobe *et al*, 1997). Blood vessels grow rapidly in tumours, are usually disorganised, often incomplete, lacking in structural integrity and prone to collapse, resulting in areas of inadequate perfusion and transient hypoxia (low oxygen tension; Blood and Zetter, 1990). Hypoxia has recently been shown to be an important stimulus for angiogenesis, promoting tumour and stromal cell secretion of such potent proangiogenic growth factors as vascular endothelial growth factor (VEGF; Bicknell *et al*, 1997). VEGF then stimulates the rapid growth of new blood vessels into hypoxic tumour areas, which results in their subsequent reoxygenation.

Tumour associated macrophages (TAMs) are widespread in human breast carcinomas (Kelly *et al*, 1988; Volodko *et al*, 1998; Lin *et al*, 2001; Bingle *et al*, 2002a) and it has been suggested that they may play an important role in the regulation of tumour angiogenesis. In *vitro* studies have shown that TAMs secrete growth factors that are mitogenic for both tumour cells and endothelial cells, in addition to stimulating the activity of a number of proteolytic enzymes in breast cancer (O’Sullivan *et al*, 1993; Pyke *et al*, 1993; Lewis *et al*, 1995; Lewis *et al*, 2000). We and others have demonstrated that breast tumours containing increased numbers of macrophages are significantly more vascularised and have greater axillary lymph node involvement than tumours with low numbers of macrophages (Leek *et al*, 1996, 1997). This could explain, at least in part, the finding that high focal TAM infiltration in primary human breast tumours is associated with reduced relapse-free and overall survival. (Leek *et al*, 1996; Volodko *et al*, 1998). A similar positive correlation between macrophage infiltration and angiogenesis has also been reported for ovarian tumours (Orre and Rogers, 1999) and B-cell non-Hodgkin's lymphomas (Vacca *et al*, 1999). Specific accumulation of macrophages expressing VEGF is seen in avascular, hypoxic areas of human breast tumours, whereas in highly vascularised, well-oxygenated areas of the same tumour, macrophages are present but VEGF is not expressed (Lewis *et al*, 2000). This suggests that hypoxia stimulates VEGF release from macrophages in tumours, and is supported by the finding that macrophages release VEGF in response to hypoxia *in vitro* (Harmey *et al*, 1998). Thus, once macrophages are located within the hypoxia/necrotic regions of the tumour, they may promote tumour progression by releasing VEGF and potentially other proangiogenic cytokines and enzymes, which stimulate tumour angiogenesis.

The mouse dorsal skinfold chamber model allows direct *in situ* visualisation and monitoring of implanted tumour spheroids and the surrounding blood vessels *in vivo* (Torres-Filho *et al*, 1995; Borgstrom *et al*, 1998; Bingle *et al*, 2002b; Nilsson *et al*, 2002). Implantation of the chamber with human breast tumour cells or spheroids permits accurate, serial, noninvasive monitoring of tumour growth, and angiogenesis using *in vivo* microscopy, facilitating accurate spatial and temporal quantification (Borgstrom *et al*, 1998; Gilead *et al*, 2004). Using this model, the requirement of VEGF for tumour angiogenesis and maintenance of vessel integrity has been demonstrated (Borgstrom *et al*, 1996; Lichtenbeld *et al*, 1998). Inhibition of endogenous VEGF results in decreased microvessel density and microvascular regression in a similar model (Jain *et al*, 1998). Moreover studies, investigating the immediate events which follow the initiation of tumour angiogenesis, provide evidence that new functional vessels form when the tumour mass consists of only 100–300 tumour cells (Li *et al*, 2000). Administration of ex-flk1 (a soluble, truncated VEGF receptor protein) resulted in partial inhibition of the early angiogenic and tumour growth activities suggesting that tumour growth is delayed prior to the onset of angiogenesis (Li *et al*, 2000).

Despite the increasing body of evidence, both experimental and clinical, implicating macrophages in breast tumour angiogenesis, there have been no reports of *in vivo* studies to demonstrate unequivocally their proangiogenic activity in tumours. The current study was designed to determine the role of macrophages in the initiation of tumour angiogenesis, by isolating the tumour spheroid from host (murine) cells. Human breast tumour spheroids were infiltrated with human monocytes *in vitro*, coated with alginate and implanted into dorsal skinfold chambers on nude mice. The resultant angiogenesis surrounding the spheroids was quantified using image analysis, and compared with spheroids consisting of tumour cells alone.

## MATERIALS AND METHODS

### Isolation of human monocytes

Peripheral blood mononuclear cells were isolated from normal healthy volunteers with Local Ethics Committee approval, LREC 02/303. Blood was collected into sodium citrate (final volume 10%) tubes and after centrifugation serum was collected and CaCl_2_ added to make platelet rich plasma-derived serum (PRPDS). The blood cells were layered onto a Ficoll gradient (Amersham Biosciences) and after centrifugation the mononuclear cell layer collected. The cells were further purified using CD14 magnetic beads according to the manufacturers instructions (Miltenyi Biotec). Briefly, the cells were incubated with the labelled beads at 4°C for 20–30 min, washed in ice-cold PBS+0.5% BSA, 2 mM EDTA, and separated from unlabelled cells using a magnetic column.

### Human breast tumour spheroids

96-well tissue culture plates (Corning) were coated with 100 *μ*l of 1.5% high-gel point agarose per well. The human T47D breast tumour cell line was subcultured and 10^4^ cells plated per well in 200 *μ*l culture medium consisting of RPMI (Sigma), 10% foetal calf serum FCS, (Sigma), 100 U ml^−1^ penicillin (Gibco), 100 *μ*g ml^−1^ streptomycin (Gibco) and 2 mM glutamine (Gibco). The plates were incubated at 37°C, 5% CO_2_ for 4 days, thereafter the culture medium was changed on alternate days by replacing 100 *μ*l of medium with 100 *μ*l fresh culture medium. Preliminary studies have established that between 20–24 days in culture the spheroids are approximately 600–800 *μ*m in diameter and have a necrotic core (unpublished results).

### Tumour spheroid coating

Preliminary studies were carried out to determine the optimum conditions for monocyte infiltration and alginate coating of the spheroids, including the assessment of VEGF and bFGF release from infiltrated and noninfiltrated spheroids. The spheroids were prepared for *in vivo* implantation using the following protocol. Spheroids were cultured for 14–18 days and human monocytes (10^5^ spheroid^−1^ well^−1^) were added and allowed to infiltrate the spheroids for 24 h. In order to prevent autologous (i.e. murine) macrophages from infiltrating the spheroid following implantation *in vivo*, the spheroids were coated with alginate (Read *et al*, 1999, 2001) by immersion in a 1.25% solution of sodium alginate (Pronova, UK) and transfer to a 0.1 M CaCl_2_ solution, prepared from a 0.15 M NaCl stock solution, to initiate gel-formation by cross-linking. The alginate coated spheroids were washed three times with PBS and once in RPMI with 10% FCS (Read *et al*, 1999, 2001). The coated spheroids were cultured for a further 5 days to allow differentiation of monocytes to macrophages. Control spheroids without monocytes were also coated with alginate and cultured in an identical manner. Monocytes alone do not form spheroids. In order to demonstrate the diffusion of macromolecules through the alginate, Hoescht 33258 (Molecular Probes) was added to the culture medium for 15 min at various timepoints up to 24 days, to allow assessment of spheroid viability and porosity over the *in vivo* time course. Spheroids were examined using a fluorescent microscope.

### Assessment of monocyte infiltration into spheroids

T47D spheroids were established in culture and allowed to grow for 17 days before purified monocytes (10^5^) were added to the culture in RPMI with 5% PRPDS, penicillin (100 U ml^−1^), streptomycin (100 mg ml^−1^) and glutamine (2 mM). The spheroids were collected on days 1, 4, and 7 after monocyte addition, formalin-fixed and embedded in paraffin. Sections were stained with anti-human CD68 (Dako, see below for immunohistochemistry details) a specific monocyte/macrophage marker, and the number of monocytes/macrophages in each spheroid section counted. For each time point at least five different spheroids and three sections from each spheroid were used to quantify the number of macrophages present.

### VEGF and bFGF quantification

The release of VEGF and bFGF critical regulators of tumour angiogenesis, were determined from spheroids both in the presence and absence of human macrophages. Culture medium was collected at various time points, before and after the addition of monocytes (days 14–18) to spheroid cultures, before and after alginate coating (days 15–19) and immediately before spheroid implantation into the skinfold chamber model (days 21–24). At all times, control spheroids (no monocyte infiltration) were cultured in parallel and medium was collected simultaneously. Immediately after collection, the medium was centrifuged to remove cell debris and stored in aliquots at −20°C until assayed. The ELISAs were performed using commercially available kits (R&D Systems) and carried out according to the manufacturers protocol.

### Histology and immunohistochemistry

Spheroids (with and without monocytes) were collected directly into buffered formalin, processed, paraffin–embedded, and cut into 5 *μ*m sections. The sections were dewaxed in xylene and absolute alcohol before endogenous peroxide was blocked with hydrogen peroxide. Antigen retrieval was achieved by incubating in protease type XXIV (Sigma) at 37°C for 20 min. After incubation in normal serum, anti-CD68 (Dako) was diluted 1 : 100 in PBS added to sections and incubated overnight at 4°C. A Vectastain kit (Vector Laboratories) was used for secondary antibody application and detection. Spheroids that had been implanted in the dorsal skinfold chamber were collected at the end of each experiment and fixed immediately in zinc chloride saline (Beckstead, 1994), processed and embedded in paraffin wax. Sections were cut as described previously, stained with haematoxylin and eosin and examined to determine the exact location of the spheroid. Appropriate other sections were stained for CD 68, (macrophages) Hypoxia inducible Factor-1*α* (HIF-1*α*, Abcam) and with anti-platelet cell adhesion molecule antibody (endothelial cells, anti-PECAM, CD-31, Abcam).

### Animals and dorsal skinfold chamber model

Adult, male MF-1 nude mice (20–30 g, *n*=10) were obtained from the University of Sheffield Field Laboratories and were allowed food and water *ad libitum*. All procedures were performed in accordance with UK legislation under Home Office Project Licence PPL 40/2343.

The dorsal skinfold chamber manufactured from titanium (Medical Workshops, University of Sheffield) was implanted into the animal under hypnorm/diazepam anaesthesia (1 : 1, 0.1 ml 100 g^−1^). The mice were placed on a heated pad, and a double layer of skin extended perpendicular to the dorsum. A circular area of skin and tissue was removed from one side to expose a single layer of striated muscle, which was then sandwiched between two symmetrical titanium frames. The tumour spheroid (approximately 600 *μ*m) was placed in close proximity to a blood vessel immediately before sealing the chamber with a glass cover slip (Lehr *et al*, 1993; Bingle *et al*, 2002b; Nilsson *et al* 2002). Angiogenesis (around spheroids and at sites within the chamber but distant to the spheroid), and associated microcirculatory variables, including vessel length, vessel number and junction numbers, were characterised using *in vivo* microscopy. Visualisation of tumour spheroid growth and angiogenesis *in situ* involved placing the mice in a plexiglass restrainer. Mice were trained to sit in the restrainer prior to surgery avoiding the need for anaesthesia and minimising any effects of stress on microvasculature function. The restrainer was fixed to the stage of a horizontally modified Nikon Optiphot microscope. The window of the chamber protruded through a longitudinal slot allowing the animal to sit in their normal position and the tumour and associated microcirculation to be viewed. Images of the preparation were monitored using a CCD camera (Hitachi, UK), displayed on a monitor (Sony PVM-1443, UK) and recorded on video (Sony S-VHS, UK) tape for off-line computerised analysis (Angiosys, UK). The day of surgery was designated day 0 and recordings were made on days 3 and 7.

At the end of the experiment the animals were killed and appropriate tissue fixed in a zinc chloride fixative before paraffin embedding, to determine the presence of macrophages in the spheroids, as previously described.

### Microvascular parameter analysis

The image analysis programme Angiosys (TCS, UK) was used to assess new vessel formation. The area immediately surrounding the spheroid and at least three randomly selected sites, distant to the spheroid, were analysed. The two groups (five animals per group) were analysed at 3 and 7 days after implantation of the spheroid; the parameters measured were mean vessel length (*μ*m), an increase indicating endothelial proliferation and differentiation, the total number of vessels per unit area (vessel density) with and without blood flow indicating the new structures are functional and the number of junctions (branch points) indicates vascular network complexity. All images were collected for analysis using a 10 × objective and the same area was measured at each time point and for each animal. A formal assessment of blood flow was not made. As the spheroids implanted in this series of experiments were coated with alginate it was not possible to measure the growth of new blood vessels into each spheroid.

### Statistical analysis

Differences between groups was analysed using the Mann–Whitney *U* test for nonparametric data. *P*-values <0.05 were considered as significant.

## RESULTS

### Monocyte infiltration of spheroids *in vitro*

**Figure 1:**
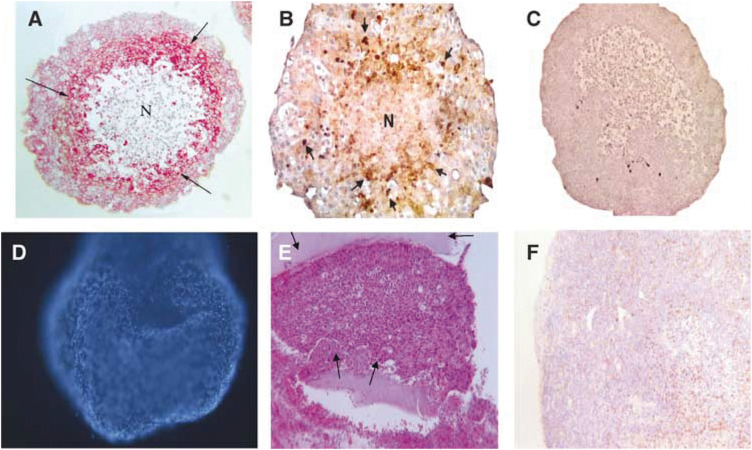
T47D spheroids cocultured with macrophages, sampled immediately before implantation and stained with (**A**) anti-CD68 antibody (brown staining, arrows; N=necrotic core) (**B**) HIF antibody (red staining, arrows; N=necrotic core) and (**C**) negative control. (**D**) T47D spheroid coated with alginate and cultured for 21 days before staining with Hoescht 33258. Under fluorescent light nuclei stain blue with this dye. (**E**) T47D spheroid collected after implantation in dorsal skinfold chamber, chamber for 7 days and stained with haematoxylin and eosin. Alginate used to coat the spheroid is clearly visible (arrows). (**F**) T47D spheroid collected in an identical manner to (**E**) but stained with anti-CD68 antibody to illustrate the continuing presence of human macrophages (brown staining).

T47D breast tumour spheroids were cultured on agarose for 15–18 days reaching approximately 600–800 *μ*m in diameter, (Figure 1A) with a hypoxic centre (Figure 1B and C) and therefore more closely mimicked a small avascular breast tumour or micrometastatic deposit. Monocytes moved into these spheroids relatively quickly, presumably attracted by various chemokines released from the tumour cells. The total number of monocytes that migrated into each spheroid was calculated at various time points after the initiation of coculture in order to determine the rate of infiltration (Figure 1A, Table 1). Monocyte migration into each spheroid continued to increase with time from 432±33.9 after 24 h to 1083±11.9 after 7 days.

The amount of VEGF released from the spheroids was measured by ELISA, and increased over the 7 days, with spheroids containing monocytes releasing at least three times more VEGF than those without monocytes (900–2000pg ml^−1^ with monocytes and 220–590pg ml^−1^ without monocytes, Table 1). An ELISA was also used to quantify bFGF release from the spheroids; however, no bFGF was detected using this method.

The major aim of this study was to investigate whether macrophages were involved in the initiation of tumour angiogenesis and thus it was necessary to isolate the tumour spheroids containing human macrophages from infiltration by mouse macrophages, which would have made data interpretation difficult. During the course of this study it was determined that the optimum method for coating spheroids, which allowed implantation into the chamber without causing damage to the spheroid, was to coculture a 14–16 day spheroid with monocytes for 24 h followed by alginate coating. The coated spheroids were cultured for a further 5 days before implantation, allowing macrophage differentiation to occur. Alginate coating the spheroids at this early time point resulted in suboptimal numbers of monocytes infiltrating the spheroid. However, further measurements of VEGF production at a time point immediately before implantation, from both infiltrated and noninfiltrated coated spheroids indicated that the macrophage-containing spheroids were releasing significantly more VEGF than the control spheroids (macrophages *vs* no macrophages 680±238 *vs* 226±75pg ml^−1^). Thus macrophages within the alginate-coated spheroid were releasing VEGF and therefore the contribution to the initiation of tumour angiogenesis could be estimated in the *in vivo* model. A homogeneous distribution of macrophages surrounding the hypoxic centre was observed in the majority of spheroid sections assessed. Three sections from five different spheroids were quantified and used to estimate the number of infiltrated macrophages per spheroid, which were approximately 400 macrophages (Table 1) at day 7.

In order to demonstrate the porosity of alginate to essential nutrients and growth factors, coated spheroids were incubated with Hoechst 33258 for 15 min, at days 10, 15, 20, and 24. Fluorescent microscopy demonstrated that at all timepoints (Figure 1D) the dye diffused across the alginate coating and stained the tumour cell nuclei.

### Angiogenesis *in vivo*

**Figure 2:**
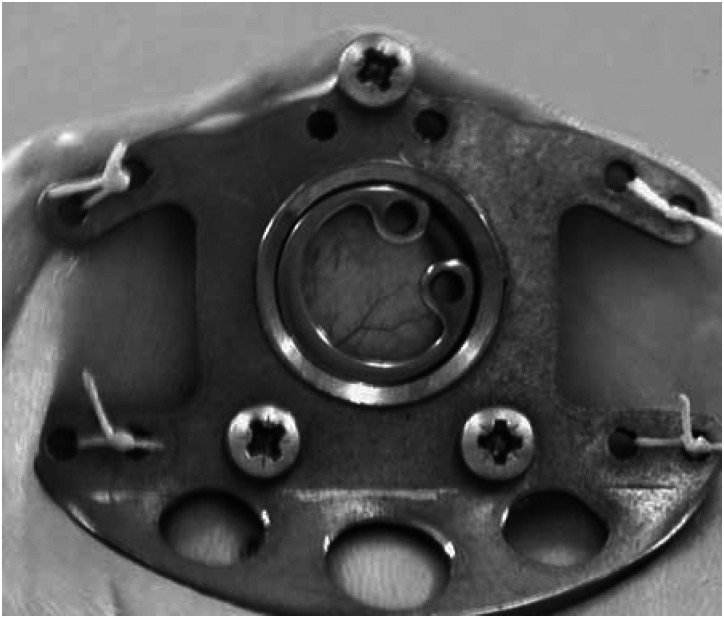
A representative photograph of the dorsal skinfold chamber implanted on a nude mouse, taken immediately after surgery. This illustrates how the chamber was attached to the animal and the lack of trauma to the tissue under examination.

The dorsal skinfold chamber was surgically implanted into nude mice, the tumour spheroids placed in the window in close proximity to a vessel at the end of surgery and covered with a glass coverslip (Figure 2). *In vivo* microscopy was performed on days 3 and 7 post-surgery as it is generally accepted that recordings cannot be made at earlier time points ensuring recovery of the surgical site from any trauma. A typical H&E stained section of a spheroid resected from the chamber at the end of an experiment, with the alginate coating clearly visible, is represented in Figure 1E. Sections were also stained for CD68 to ensure the macrophages were still present but the number of macrophages in each spheroid was not assessed at this time (Figure 1F). The sections were also stained with F480, a specific marker of murine macrophages, but no positive staining was detected (data not shown).

**Figure 3:**
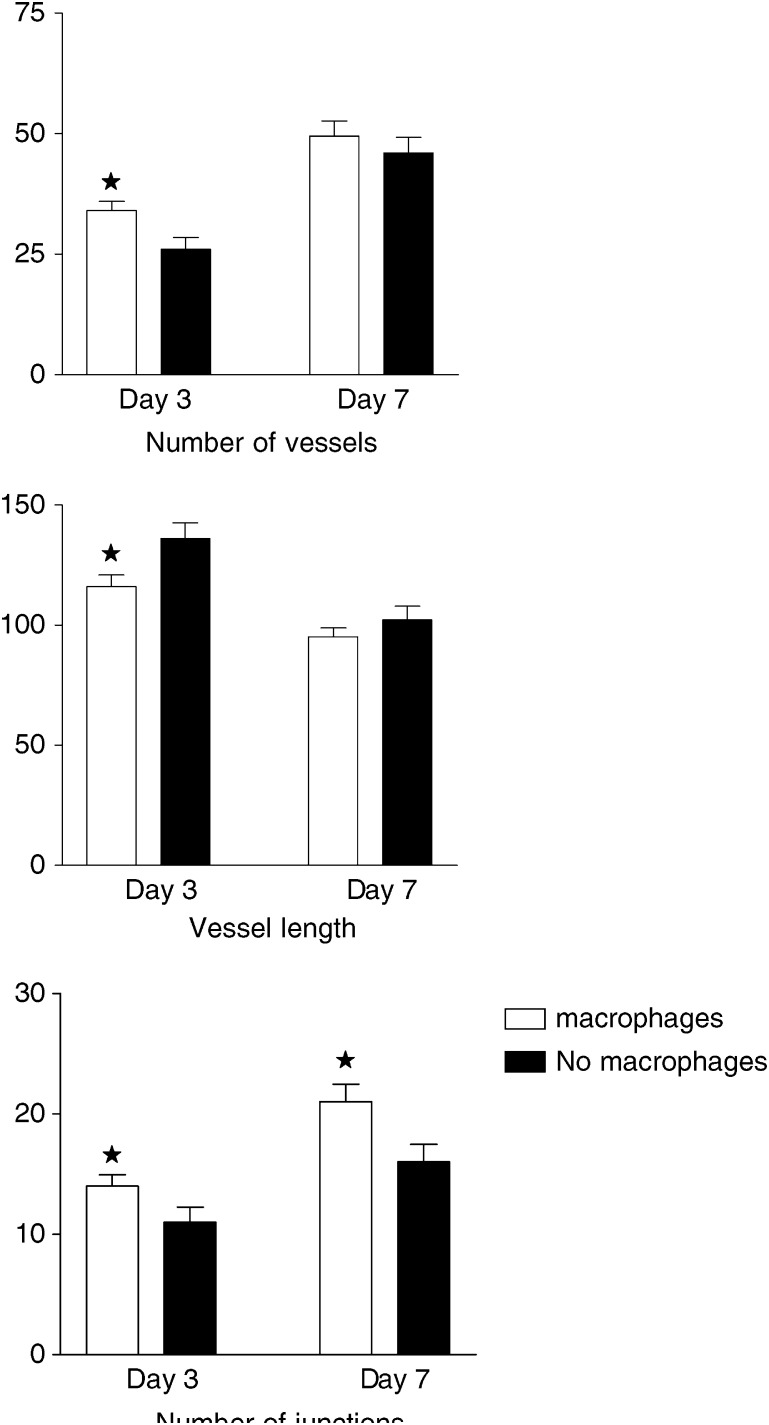
Angiogenesis was assessed off-line from video recordings taken three and seven days after implantation of alginate coated breast tumour spheroids. The image analysis package Angiosys, UK was used to quantify vessel length, the number of vessels and the number of junctions surrounding spheroids with and without macrophage at days 3 and 7 after implantation. (*n*=5 mean±s.e.m.). ^*^ Denotes *P*<0.01(vessel number, day3), 0.008 (vessel length day 3), 0.025 (number of junctions day 3), 0.021 (vessel length day 7) when compared to no macrophage group using Mann–Whitney *U* Test.

The data collected from off-line analysis indicates that angiogenesis were stimulated by the breast tumour spheroids in the presence or absence of macrophages (Figure 3) but the stimulus was increased in the early phase of angiogenesis (day 3) if macrophages were present. Although the data showed that breast tumour spheroids stimulated angiogenesis in the absence of macrophages it also showed that the stimulus was greater in the early phase of tumour angiogenesis if macrophages were present (Figure 3). There was a significant difference between those spheroids which had been infiltrated with human macrophages, for all parameters measured on day 3; increased vessel number *P*<0.011, increased number of junctions *P*<0.025 and shorter mean vessel length *P*<0.046 all indicating increased angiogenesis in the presence of macrophages. By day 7 the number of vessels was significantly greater than on day 3 (49.5±3.08 and 34±1.95 *P*<0.001 with macrophages, 46±3.29 and 26±2.47 *P*<0.001 without macrophages) as was the number of junctions (21±1.46 and 14±0.93 *P*<0.001 with macrophages, 16±1.47 and 11±1.25 *P*<0.002 without macrophages) implying that new vessels had branched from existing vessels. The mean tubule length measured at these times was less on day 7 than on day 3 (95.2±3.58 and 116±4.92 *P*<0.001 with macrophages, 102.2±5.58 and 136±6.52 *P*<0.002 without macrophages) indicating that new shorter blood vessels had sprouted due to the stimulatory effects of the tumour spheroid. However, by day 7 the differences between spheroids with and without macrophages were no longer significant. (Figure 3) A formal assessment of blood flow velocity was not made but the video recordings indicated that the vessels measured were functional.

## DISCUSSION

This is the first study to demonstrate that macrophages may modulate tumour angiogenesis in the early stages of development, with increased number of vessels and branches. A number of studies have shown that macrophages congregate in large numbers in the hypoxic areas of many solid tumours, which may be due to the influence of hypoxia on the chemoattractant signalling cascade in the tumour microenvironment ((Negus *et al*, 1997; Ueno *et al*, 2000, Grimshaw and Balkwill 2001). Macrophages have the ability to both positively and negatively regulate tumour growth (Mantovani *et al*, 1992); tumour cell cytostasis may be induced, but in contrast tumour cell survival may be promoted by macrophage activation and release of angiogenic and mitogenic cytokines in the tumour microenvironment, such as VEGF (Barbera-Guillem *et al*, 2002), pro-inflammatory cytokines and enzymes (Kataki *et al*, 2002; Blot *et al*, 2003). A recent *in vivo* study by Wyckoff *et al* demonstrated a synergistic relationship between breast tumour cells and macrophages in cell migration and suggested a role for tumour associated macrophages in metastasis (Wyckoff *et al*, 2004). In addition clinical studies in breast and endometrial cancer have reported positive correlations with angiogenesis and/or survival (Leek *et al*, 1996; Salvesen and Akslen, 1999). However, the precise role of monocytes and macrophages in tumour angiogenesis and the growth of solid tumours is as yet unclear.

In the current study human monocytes isolated from peripheral blood were cocultured with human breast T47D tumour spheroids allowing monocyte migration into the tumour spheroid where differentiation into tumour-associated macrophages occurs (Konur *et al*, 1998). A necrotic core developed in the breast tumour cell spheroids presumably due to the hypoxic central regions, with HIF1*α* staining evident surrounding the necrosis. This was similar both in the presence and absence of macrophages and therefore is unlikely to be due to a direct anti-tumour action of the macrophages. VEGF is a potent endothelial cell mitogen and has previously been shown to be released from both T47D tumour cells and macrophages (Blancher *et al*, 2000) Breast tumour spheroids containing macrophages demonstrated elevated VEGF release (determined by ELISA) when compared to spheroids cultured in the absence of macrophages. In addition a second angiogenesis factor bFGF was measured, using an ELISA, but release of this mitogen was not detected. This data is in agreement with previous studies demonstrating that T47D human breast tumour spheroids/cells do not release bFGF (Luqmani *et al*, 1992) The aim of the current study was to identify whether tumour associated macrophages are involved in the initiation of angiogenesis. Thus the spheroids required coating to allow diffusion of angiogenic factors out of the spheroids but prevent spheroid infiltration of autologous murine macrophages. A number of studies have used alginate, derived from brown seaweed, as a means of encapsulating cells for *in vivo* implantation, thereby isolating them from the host immune cells. Previous studies demonstrated the coating has no effect on cellular growth or proliferation, and indicated that in the first week after implantation *in vivo*, mononuclear cells migrated to the rim of the alginate spheroid but there was no penetration, and over the following nine weeks, the number of migrating cells gradually decreased (Read *et al*, 1999, 2001). The permeability to diffusible molecules was demonstrated by Hoescht staining of tumour cells, following addition to the culture medium, and the release of VEGF but not cells into the well suggesting that coating allowed the passage of molecules but not cells into and out of the spheroids. Immediately before implantation a sample of spheroids was collected, and processed for histology and immunohistochemistry, which demonstrated the viability of both tumour cells and macrophages, surrounding the hypoxic centre. The number of macrophages present in breast tumour samples has rarely been documented, the majority of published studies relating tumour associated macrophages with tumour survival focus on macrophage “hot spots” and their relationship to markers of angiogenesis in tumour sections. A recent study by Bouma-ter Steege *et al* (2004) did, however, calculate the relative levels of leukocytes within breast tumour biopsies and found 490 macrophages/mm^2^ in medullary carcinomas and 343 mm^−2^ in ductal carcinomas.

The role of macrophages has not been extensively studied *in vivo*. The hypothesis of the current study is that the presence of macrophages increase the angiogenic potential of tumour spheroids through the increased production of VEGF. Thus to evaluate any role for macrophages in the initiation of tumour angiogenesis, an *in vivo* model involving the implantation of breast tumour spheroids into a dorsal skinfold chamber on nude mice has been developed in our laboratory. The mice are immunodeficient, with an absence of T cells but a normal complement of B cells and elevated levels of both natural killer cells and macrophages. Macrophages in nude mice appear more potent than those from mice with a normal thymus (Budzynski and Radzikowski, 1994).

Following implantation, parameters measured to determine any effect of the macrophages on angiogenesis included vessel length, vessel numbers, junctions or branch points, in areas directly adjacent to, in addition to distant from the spheroid. Mean vessel diameter is not presented as often distant sites included large host vessels present throughout the procedure, which skewed analysis. The angiogenesis parameter measurements three days after implantation of the tumour spheroid indicated that macrophages play a role in the initial angiogenic response, with significant increases in the density of blood vessels and the number of junctions, with a reduction in vessel length in comparison to control spheroids composed solely of tumour cells. The shorter vessel length was an unexpected and as yet unexplained finding although vessels were generally of a length found in other *in vivo* models (Donovan *et al*, 2001). Tumour cells are a known source of angiogenic factors and T47D tumour cells produce VEGF, thus it was not surprising that over the course of the experiment, the tumour spheroids without human macrophages showed evidence of increased angiogenic activity surrounding the spheroids. By day 7 there were no significant differences in the angiogenic parameters measured between the experimental and control groups, although immunohistochemistry demonstrated that the macrophages continued to release VEGF. At the end of each experiment all tissue from the chamber was processed and stained, and demonstrated that the tumour cells remained viable, that macrophages remained within the spheroid throughout the experimental period and that host macrophages had not permeated the alginate coating. The size of each spheroid was not measured at the end of the experiments, but previous studies have shown that it is possible for cell growth to occur within the confines of the agarose coating (Read *et al*, 1999, 2001).

In conclusion therefore, the current study demonstrates that macrophages present within a solid tumour may significantly contribute to the initiation of angiogenesis. However, in the absence of macrophages, tumour cells produce the necessary stimuli to initiate tumour angiogenesis but initiation is delayed. Further long-term studies, using a syngenic murine model and uncoated spheroids infiltrated with host murine macrophages, allowing the tumour stroma to play a role, or with host macrophage function inhibited, would allow the contribution of macrophages to the maintenance of angiogenesis, endothelial cell survival and tumour growth to be determined. In addition the model could be used to assess the future of the macrophage as a target for tumour therapy.

## Figures and Tables

**Table 1:** The number of monocytes/macrophages infiltrating the spheroids was assessed by counting CD-68 positive cells present in representative sections

	**Spheroids with macrophages**			**Spheroids without macrophages**		
	**Day 14**	**Day 18**	**Day 21**	**Day 14**	**Day 18**	**Day 21**
Number	432±34	667±39	1083±12			
VEGF (pg/ml)	900±51^*^	1350±85^*^	2000±123^*^	220±15.3	450±25.2	590±26.7

The spheroids were sampled at the time points indicated. VEGF (pg/ml) secreted by the uncoated spheroids in the presence and absence of monocyte macrophages is presented. All data are presented as mean±s.e.m. and ^*^denotes statistical significance *P*>0.05 compared to control.
